# Social gaze dynamics in teams: Comparing face-to-face and video meeting settings

**DOI:** 10.1371/journal.pone.0329060

**Published:** 2026-03-02

**Authors:** Petra Nieken, Tom F. Reuscher

**Affiliations:** KIT Department of Business and Economics, Institute of Management, Karlsruhe Institute of Technology, Germany; CNRS: Centre National de la Recherche Scientifique, FRANCE

## Abstract

Understanding the factors that promote cooperative behavior in teams is essential for organizational success. While the role of verbal communication in fostering cooperation and team cohesion is well established, the impact of nonverbal signals, particularly eye contact and related patterns, known as social gaze dynamics, has been neglected. In this study, we conducted a multiparty eye-tracking experiment, enabling us to explore the relationship between social gaze, cooperation, and team cohesion. In particular, teams of three participants engaged in a two-stage task: a structured pre-play communication stage (1) and a social dilemma game (2). During pre-play communication, eye-tracked participants performed a real-effort task mirroring the ensuing social dilemma. This allowed us to capture social gaze dynamics and assess their relation to subsequent cooperation and team cohesion. Specifically, we compared the emergence and impact of attentional reciprocity between face-to-face and video meeting settings and provided initial evidence that the extent to which direct gaze is reciprocated within teams influences subsequent cooperative behavior. Overall, our findings highlight potential downsides of increasingly shifting team meetings to the virtual space, where the absence of gaze information affects the emergence and impact of social gaze dynamics.

## Introduction

In the modern workplace, organizational success often depends on team work. However, the conflict between prioritizing individual gains and maximizing collective benefits can significantly challenge the productivity and success of a team [1–4]. To mitigate this issue, it is crucial to understand the factors that drive cooperative behavior. Previous research has largely focused on verbal aspects of team communication, such as commitments and agreements, while neglecting the potential power of nonverbal signals (for reviews, see [5,6]). In this study, we address this research gap by examining the role of interdependent attentional processes within teams, known as social gaze dynamics [7,8].

Specifically, we investigate the impact of social gaze dynamics on cooperation and team cohesion across two commonplace modes of team communication: face-to-face meetings and video meetings. Therefore, we conducted a controlled laboratory experiment in which teams of three participants engaged in a two-stage team task: a structured pre-play communication stage followed by a social dilemma game [9]. During the pre-play communication stage, eye-tracked participants discussed their contribution to a real-effort team task. In the second stage, they decided on their costly contribution in private. In particular, we employed a multiparty eye-tracking setup in the communication stage to measure each team member’s eye movements synchronously, allowing us to capture social gaze dynamics within a naturalistic team setting (1) and relate them to the team output (2).

Recent studies have shown that individuals can intuitively assess others’ choices and prosocial motives by observing their gaze [10,11]. The core idea of these studies is to make the gaze patterns visible in a two-player game to investigate whether gaze information conveys information about intentions and strategies and can therefore also be used strategically. By design, the gaze information in these studies was very salient (e.g., dynamic gaze focus mapped onto a payoff matrix). In contrast to these studies, we developed a novel multiparty eye-tracking set-up to study gaze patterns in a discussion about the ensuing task. We abstain from visualizing the gaze patterns of participants because our goal was to study the role of social gaze dynamics that are naturally emerging during face-to-face and video meetings.

The experiment encompassed two treatments. In the first treatment, teams participated in a face-to-face meeting in the pre-play communication stage. In the second treatment, they communicated in a video meeting. While face-to-face meetings encompass the whole spectrum of nonverbal signals (e.g., gaze, body language, and gestures), conventional video meeting systems are not capable of conveying what others are visually attending to. By comparing these two treatments, which differ in the informative value of gaze (e.g., whether a person is gazing at the task or another team member), we were able to study differences in the emergence of social gaze dynamics between face-to-face and video meeting settings and their impact on subsequent cooperation and team cohesion.

Our results revealed that team members reciprocate the direct gaze of other team members significantly more frequently in face-to-face than in video meetings, resulting in higher levels of attentional reciprocity overall. Furthermore, we found that the extent to which team members reciprocated each other’s direct gaze in face-to-face meetings significantly enhanced overall cooperation. However, in video meetings, the ambiguity of gaze information eliminated this positive effect, raising concerns about increasingly shifting team communication to the virtual space.

Our paper is related to the literature investigating the impact of communication on team cooperation and team cohesion in general and the impact of gaze on decision-making in particular. In the following, we discuss these strands of literature and highlight our contribution.

### Communication in teams

Team work and other social dilemmas share the problem of free-riding and social loafing. The underlying economic phenomena have been studied extensively [9,12–14]. There is ample evidence that communication helps to overcome these issues in team cooperation. Specifically, the opportunity to communicate has been shown to increase cooperation rates in social dilemma games by 40% on average (for reviews, see [5,6]). In an attempt to reveal the key mechanisms of the communication effect, [15] identified two crucial factors: verbalized commitments (e.g., agreements) and type detection, the ability to infer others’ cooperativeness by interpreting more subtle verbal and nonverbal signals. While verbalized commitments promoted cooperation by highlighting social norms, type detection allowed individuals to discern cooperative from less cooperative team members and adapt accordingly. The authors showed that the largest fraction of the positive effect of communication could be attributed to type detection (73.3%). In addition, the positive effect of communication increases with group size and is particularly pronounced when communication takes place in face-to-face meetings [5,16]. further investigated whether the channel of communication affects cooperative behavior. They found that the positive impact of communication persists in remote settings but depends on the degree to which a communication channel resembles the nonverbal richness of face-to-face communication. In particular, communication via video meetings has been shown to produce significantly higher and more stable cooperation rates than text-based chats and audio-based channels [17,18]. More broadly, the literature has established that communication modalities shape important economic outcomes, including honesty [19], leadership [20], and creativity [21,22]; for a review, see [23]). Overall, these findings suggest that, in part, cooperative behavior depends on the richness of nonverbal signals, such as gaze information that allows individuals to gather information about the type of the other team members.

### Social gaze dynamics

The role of social gaze dynamics has been studied extensively in various disciplines such as biology, psychology, and, recently, economics. Previous research has shown that human faces, especially the eyes, automatically attract visual attention [24,25]. Eye movements serve both a perceptual and a communicative purpose—a concept known as the dual function of gaze [26]. In social interactions, gaze patterns that involve multiple individuals are conceptualized as social gaze [7,8]. Its three core dynamics are *mutual gaze*/ (i.e., individuals are directly gazing at each other), *gaze aversion* (i.e., an individual gazes at another who is gazing somewhere else), and *joint attention* (i.e., individuals are gazing at the same object or aspect within the visual scene).

In face-to-face settings, prolonged episodes of mutual gaze have been shown to be positively correlated with desirable factors [27], such as trust, rapport building, and establishing common ground between individuals (for a literature review, see [28]). In contrast, gaze aversion has been linked to adverse outcomes. For example, [29] observed a negative correlation between gaze aversion and the quality of agreements in dyadic negotiations. Consequently, social gaze plays an important regulatory role in face-to-face communication and may shape economic behavior when working together in teams.

The study of gaze has gained popularity in behavioral economics because it provides insights into underlying cognitive processes [30]. In particular, eye-tracking has been used to understand how visual attention relates to economic behavior. For instance, previous research has shown that cooperative behavior in several economic games, such as money allocation tasks, public goods dilemmas, and sender-receiver games, can be explained through a post-hoc analysis of individuals’ gaze during information acquisition [31,32]. These studies demonstrate that observing a counterpart’s gaze could allow individuals to derive beneficial insights, potentially enhancing their strategic decisions. As the feeling of being observed can influence an individual’s behavior, one might worry that the usage of eye-tracking alters economic behavior. However, [33] found no considerable changes in eight standard economic games.

Extending this line of research, recent studies have used eye-tracking as an interactive tool, allowing participants to observe their counterpart’s gaze during decision-making in real time. For instance, [11] revealed that the option to observe gaze enabled participants to infer their counterpart’s hidden choices in a series of sequential 2x2 box choice games. Moreover, when the eye-tracked participants knew that their gaze was transmitted, they strategically adapted it during decision-making to facilitate coordination. A related study examined whether (visualized) gaze information could help participants identify their counterpart’s prosocial motives in a money allocation task [10]. The authors found that participants who observed the allocators’ gaze were able to judge their prosocial motives. However, when allocators were incentivized to appear more prosocial, they strategically adapted their gaze, making it more difficult for observers to accurately predict their hidden choices. Taken together, these studies show that gaze functions as a strategic signal, enabling individuals to infer others’ choices and prosocial motives. While these experimental setups offer precise control for studying gaze behavior in laboratory settings, they rely on displaying gaze patterns on a computer screen. In contrast, our multiparty eye-tracking setup allowed us to examine the emergence of social gaze dynamics in a natural team setting.

### Team cohesion

In addition to the profound effect of communication on cooperative behavior, a large body of literature revealed a close link between team cohesion and cooperative behavior [2,34]. Team cohesion is a multifaceted construct that has consistently been shown to have a large and robust positive effect on team performance [35–37]. According to [38], team cohesion includes several subdimensions of which the most pertinent are belongingness (i.e., the degree to which team members are attracted to each other), social cohesion (i.e., the closeness of social relationships), and task cohesion (i.e., the bonding between team members based on shared objectives). Despite its multifaceted nature, prior research has predominantly focused on isolated dimensions of team cohesion. For example, [2] investigated the dimension of social cohesion. In contrast, [38] suggested capturing team cohesion according to the belongingness dimension. Their findings align with previous research, showing that high-cohesion teams are significantly better at coordinating on superior equilibria in strategic situations. In addition, the authors found that team cohesion increases cooperation primarily by shaping beliefs about the willingness of other team members to cooperate.

Taken together, these findings suggest that successful cooperation is significantly influenced by the level of team cohesion as well as the channel of communication, which determines how much information can be exchanged within teams. Our study extends these strands of literature by examining whether social gaze, as one specific aspect of nonverbal communication, affects cooperation and team cohesion.

## Materials and methods

The study was preregistered at AsPredicted (#130545) and approved by the ethics committee of the Karlsruhe Institute of Technology (KIT). The individuals displayed in this manuscript have given written informed consent (as outlined in PLOS consent form) to publish their case details. In the following, we describe the general study setup, treatments, and procedures. We also provide information on our sample, hypotheses, and the eye-tracking technology used for this study. Overall, the study included a two-stage team task and two brief surveys. We programmed the team task in oTree (v5.0.0a21; [39]) and deployed it via Heroku (v22.0; [40]). We used SoSci-Survey (v3.4.00; [41]) to elicit personal survey data. The treatments varied only in the first stage of the team task, with all other procedures held constant.

### Participants

Participants were recruited from the KD2Lab subject pool using Hroot [42]. Participation in the 45-minute experiment required German language skills at the native-speaker level and unrestricted or appropriately corrected vision and hearing. Furthermore, wearing glasses was defined as an exclusion criterion, because head-mounted eye-tracking devices had to be worn during the study. The participants received an endowment of EUR 7.20 and could receive additional earnings of up to EUR 15.30. Overall, the average payment was EUR 13.69.

The required sample size of 68 teams (204 participants) was calculated with G*Power (v3.1.9.7; [43]), using an alpha error probability of 0.05, a statistical power of 0.80, and assuming a medium effect size (f² = 0.15). Overall, we collected the data from 77 teams since we had to drop five teams due to technical issues and another four teams because they contained at least two members who knew each other before participation. Finally, we analyzed the data from the 68 teams, including a total of 204 participants aged between 18 and 54 years (*M* = 24.4, *SD* = 4.85). Gender-homogenous teams were randomly assigned to the two treatments, resulting in 34 teams each.

### Procedure

In all treatments, the participants went through four distinct phases with a total duration of 40 minutes. Each experimental session consisted of three participants of the same gender forming a team. We decided to maintain gender homogeneity to minimize confounding effects on team communication that could arise in heterogeneous teams [44]. At the beginning of each session, participants sat in a private cubicle and completed a brief survey capturing demographic information, dispositional collective orientation [45,46], interpersonal trust [47], and personality traits [48]. In the second phase, participants received the full instructions regarding the team task, payoff structure, and session outline (for detailed instructions, see Supporting Information S1 File). Next, they entered the first stage of the team task which was a five-minute pre-play communication stage. After the pre-play communication stage, we guided them back to their individual cubicles. In the second stage of the team task, participants made their payoff-relevant decisions in private. They knew that they would not meet the other participants again. After making their decision, they filled out a short survey eliciting self-reported team cohesion [49–51], social presence [52], and familiarity between team members.

### Team task & multiparty eye-tracking

**Fig 1:**
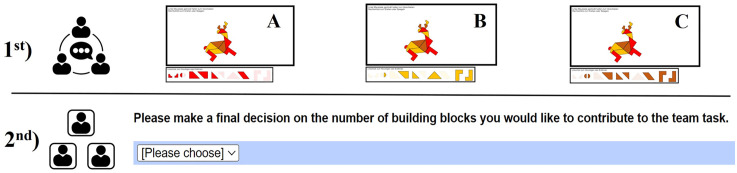
Two-Stage Team Task. Pre-play communication stage in teams (top) and the second stage with the binding contribution decision in private (bottom).

To investigate the relationship between social gaze dynamics and cooperative behavior, we designed a team task involving three participants per session. The team task comprised two stages: a structured pre-play communication stage and a social dilemma game, drawing on the model by [9]. Specifically, the pre-play communication stage was structured around real-effort task that mirrored the key elements of the ensuing social dilemma game (Fig 1). During this initial stage, we employed a multiparty eye-tracking setup to synchronously measure each team member’s eye movements. Therefore, we equipped the participants with Pupil Invisible eye-tracking glasses [53] and led them from their individual cubicles to a separate room. The head-mounted devices did not restrict the participants’ freedom of movement as they feature an unobtrusive, lightweight design. While recording, the eye-tracking glasses were connected to mobile phones (Pupil Labs Companion Device; OnePlus 8) via a single USB-C cord to calculate gaze data in real-time at a sampling rate of 200 Hz (Pupil Labs Companion App; v1.4.23). Further computations to prepare the eye-tracking data for statistical testing were performed in R [54].

This approach was essential as it allowed us to capture social gaze dynamics within a natural team setting, which related to the level of cooperation in the subsequent stage. Additionally, we chose to collect eye movements during free-form communication because “gaze should not be treated as an isolated phenomenon, but as one aspect of the interaction, which is multimodal by nature” [28]. In the following, we provide a detailed description of both stages.

#### Stage 1: pre-play communication with real-effort task.

To encourage vivid communication between team members, we used a real-effort task with a creative element. The task was to create a tangram, a puzzle composed of geometric pieces that can be arranged to create various shapes reminiscent of animals or objects. Tangrams are frequently employed in eye-tracking studies as collaborative problem-solving tasks because they require close cooperation and visual coordination among participants [55,56].

In our setup, each team member could contribute up to twelve pieces to build a unique shape. Importantly, this stage served solely as a communication phase, not a payoff-relevant decision. The purpose of the task was to create a context for an open discussion and exchange.

The participants knew that the number of pieces used in this stage would not affect their actual payoff and that they would later make their true, incentivized decision in private. Nevertheless, the payoff structure was common knowledge in Stage 1, allowing participants to use the tangram game as a means to signal intentions and make verbal or visual agreements on how much to contribute. Thus, Stage 1 functioned as a non-binding (cheap-talk) phase, designed to mirror real-world team interactions in which individuals discuss their intended contributions before executing a task.

#### Stage 2: payoff-relevant decision in private.

After completing the pre-play communication stage, participants proceeded to the second stage, where they made their payoff-relevant contribution to the team task in private.

To induce a social dilemma, contributing to the team output was costly. Each piece $x_{i}$ led to costs given by, $c_{i}=c(x_{i})=x_{i}^{\text{2}}$. The revenue of the team was given by R$\;=\text{1}\text{8}*\sum_{i=\text{1}}^{\text{3}}x_{i}.\;$ The revenue was split equally among all team members. Thus, a risk-neutral, money-maximizing participant would choose to contribute 3. However, the total revenue is maximized if each team member contributes 9. Accordingly, contributions of more than 3 pieces were an indicator of cooperative behavior in our set-up.

Importantly, decisions made in this stage were independent of the tangram pieces used or discussed in Stage 1. Participants were free to deviate from any previously stated commitments.

### Treatments

**Fig 2:**
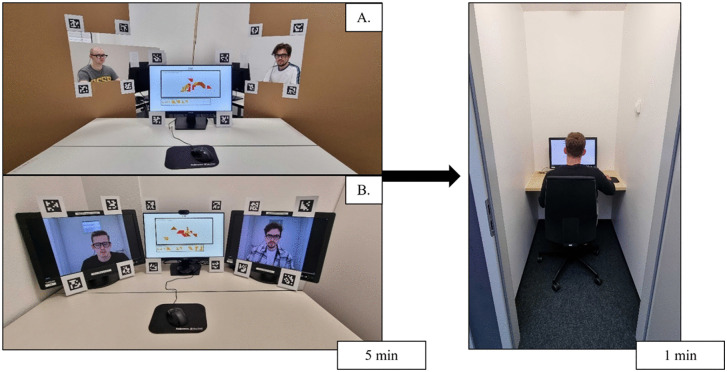
Experimental Setup. Pre-play communication stage from team member A’s perspective in face-to-face (A) and video meeting setting (B; left), and the second stage in an isolated cubicle (right). The individuals in this figure have given written informed consent (as outlined in PLOS consent form) to publish their case details.

Gender-homogenous teams were randomly assigned to one of two treatments. These treatments only altered whether pre-play communication in the first stage of the team task took place in a *face-to-face meeting* (*FT*) or in a *video meeting* (*VT*). In both treatments, team members could see and verbally communicate with each other. The treatments manipulated only whether team members could discern where each other was visually attending to, without altering the access to other verbal and nonverbal signals (Fig 2).

#### Treatment 1: Face-to-Face (FT).

For the pre-play communication stage in *FT*, participants were led to a group room and then asked to take a seat at individual desks equipped with a computer and monitor to perform the tangram task together. The desks were separated from each other by three partitions. The partitions contained rectangular cut-outs so that participants were able to see and verbally communicate with each other. To be as close as possible to *VT*, the cut-outs made sure that the participants could only see the upper part of each other.

#### Treatment 2: Video Meeting (VT).

In *VT*, participants were led to individual rooms for the pre-play communication stage. In these rooms, they were asked to sit at a desk equipped with a computer and three monitors. While performing the team task on the central monitor, team members were displayed on the other two monitors so that the participants were able to see and verbally communicate with each other despite being spatially separated. We used a locally hosted instance of BigBlueButton (v2.5) to set up the video meetings.

### Data preparation & key variables

Our key variables were team output, self-reported team cohesion, and social gaze dynamics at the team level. Below, we describe how these variables were defined and prepared the data for statistical analyses.

#### Team output.

To capture cooperative behavior in the team task, which involved the trade-off between individual and team payoffs, we measured team output as the total amount of individual contributions to the team task*.*

#### Team cohesion.

Following the multidimensional conceptualization by [38], we operationalized team cohesion by combining three scales capturing belongingness (IOS; [49]), social cohesion (GCS; [50]), and task cohesion (GEQ; [51]). We calculated the unweighted mean across these scales for each participant and then aggregated the scores at the team level.

#### Social gaze dynamics.

We quantified social gaze dynamics, *mutual gaze*, *gaze aversion*, and *joint attention* using a multi-step process. First, we used the open-source software Pupil Player (v3.5.1; [53]) to assign each participant’s gaze points during the pre-play communication stage to one of the following areas of interest (AOIs): A, B, C, and Task (see Supplementary Information: S4 Fig). To ensure reliable AOI tracking despite participants’ head movements, we affixed QR codes to the corners of each AOI. Pupil Player’s built-in image recognition feature automatically detects these QR codes and dynamically maps gaze points to AOIs, ensuring robust eye-tracking under naturalistic conditions. The AOIs A, B, and C refer to the respective team members displayed on the monitors to the left and right of each participant (e.g., C could gaze at team members A and B). The AOI Task referred to the central monitor on which the team task was performed. The remaining gaze points outside these areas were classified as Else.

**Fig 3:**
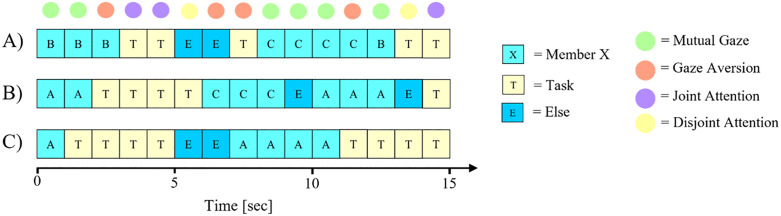
Operationalization of Social Gaze Dynamics. Exemplary time series of three team members with assigned areas of interest per interval and corresponding social gaze dynamics.

Gaze data was then processed in R [54]. First, each participant’s time series of gaze points to the defined AOIs was cut into sequences, corresponding to the exact duration of the pre-play communication stage. Time series were synchronized between team members by using an annotation cue at the beginning and end of the pre-play communication stage. Next, we cut each time series into 100-millisecond slices. Subsequently, each slice was assigned the AOI that received the most gaze points during that period. Due to the high sampling rate of 200 gaze points per second, the described aggregation procedure was robust against missing values. Following the framework by [29], we categorized every unique combination of AOIs to one of the conceptualized social gaze dynamics: *mutual gaze*, *gaze aversion*, *joint attention*, and *disjoint attention* (see Fig 3).

- *Mutual Gaze*: Two team members gaze at each other simultaneously (e.g., A_B_ ∧ B_A_ ∧ C_A_).
- *Gaze Aversion*: At least one participant gazes at a team member who is not reciprocating and no mutual gaze is present (e.g., A_B_ ∧ B_Task_ ∧ C_Else_).
- *Joint Attention*: All team members gaze at their task monitors simultaneously (A_Task_ ∧ B_Task_ ∧ C_Task_).
- *Disjoint Attention*: Remaining AOI combinations were defined as *disjoint attention* (e.g., A_Task_ ∧ B_Else_ ∧ C_Else_).

To calculate the proportion to which *mutual gaze*, *gaze aversion*, and *joint attention* occurred, we divided the number of slices assigned to each social gaze dynamic by the total number of slices per team. Finally, we computed the level of *attentional reciprocity* within teams as the ratio of *mutual gaze* to the number of cases in which at least one participant gazed at another team member (i.e., the sum of *mutual gaze* and *gaze aversion*). *Attentional reciprocity*—referred to as Mutual_Gaze_Ratio in the preregistration but renamed in the paper for improved clarity without altering its meaning or measurement—is operationalized as the extent to which gazing at other members during the pre-play communication stage resulted in *mutual gaze* rather than *gaze aversion* ($\frac{Mutual\;Gaze}{Mutual\;Gaze\;+\;Gaze\;Aversion}$).

Importantly, in our study mutual gaze is defined as an objectively measurable state of visual attention—that is, two team members simultaneously directing their gaze at each other. Such states could occur in both treatments (*FT* and *VT*) and be detected behaviorally. However, only in *FT* could these episodes also be recognized by the participants themselves, because gaze was directly visible and perceptually accessible. Team members could clearly discern where others were looking and notice when their gaze was returned. In contrast, the *VT* introduced ambiguity inherent to state-of-the-art video meeting systems. Participants viewed team members on side screens while attending to the shared task on a central screen, with the webcam placed above that central monitor. Looking into the camera to simulate eye contact meant not looking at the side screens, making it impossible to both maintain visual attention to team members and produce the appearance of direct gaze. Although participants could infer head orientation, they could not reliably determine whether another team member was attempting to engage in mutual gaze with them or with someone else. Thus, even when an episode of mutual gaze occurred in *VT*, it was unlikely to be experienced as such by the participants. This perceptual asymmetry is central to our treatment design: while *mutual gaze* as a behavioral state was equally measurable in both treatments, only in *FT* was it perceptually accessible. The treatments therefore manipulated not the possibility of mutual gaze itself, but the option to perceive mutual gaze and, with it, attentional reciprocity.

While our operationalization follows established computational paradigms and ensures consistency across the treatments, it is important to acknowledge the limitations of this approach in the *VT*. The measure does not guarantee that the team member experienced the perpetual equivalent of mutual gaze. Given the position of the webcam and the screens, it was technically impossible to look at a team member and simultaneously appear to be looking into the camera. Thus, our measure in *VT* reflects coordinated attention towards social stimuli, but not necessarily the felt sense of being seen. This distinction is a known feature in screen-mediated interactions and underscores the importance of studying gaze dynamics.

### Main hypotheses

Previous research has shown that communication is pivotal for successful cooperation in teams [5,6,19,57,58]. In particular, [15] observed that individuals evaluate a variety of verbal and nonverbal signals to detect their team members’ social type and adapt their economic behavior accordingly. Consistent with this, several studies have found that the mode of communication influences cooperation in social dilemmas because the given nonverbal richness determines the amount of information that can be exchanged between team members [16].

Building upon these findings, we aimed to understand the underlying mechanisms leading to differences in cooperation between two commonplace modes of team communication: face-to-face and video meeting settings. One of the most distinguishing factors between these settings is the ambiguity of gaze in the latter. While face-to-face communication encompasses the full range of nonverbal signals, conventional video meeting systems do not inform individuals about their team member’s focus of gaze. Given that virtual team members cannot recognize whether they are gazing at each other, we expected that the coordination of gaze is hampered in *VT*, resulting in relatively lower extents of *mutual gaze* as well as relatively higher extents of *gaze aversion* as compared to *FT*. Accordingly, our first hypothesis posited that the level of *attentional reciprocity*, defined as the extent to which looking at others during the pre-play communication stage resulted in *mutual gaze* rather than *gaze aversion*, would be higher in *FT* than *VT*.

**H1.** *The level of attentional reciprocity within teams is higher in FT compared to VT.*

Several studies revealed that the level of *mutual gaze* is linked to positive outcomes in social interactions, whereas *gaze aversion* is associated with negative outcomes [27,29,59,60]. In addition, recent research indicates that individuals understand gaze information as a signal for prosocial motives and adapt their economic behavior accordingly [10,11]. Thus, our second hypothesis proposed that higher levels of *attentional reciprocity* positively affect cooperative behavior, measured as *team output*.

**H2.** *Higher levels of attentional reciprocity within teams positively affect team output.*

Moreover, previous research suggests that the perception of *mutual gaze* increases the probability of memorizing others, and makes individuals feel more attracted to others than *gaze aversion* [61,62]. Similarly, [63] claimed that the ambiguity of gaze information in video meetings interferes with the processing of nonverbal signals and thus hinders the formation of social relationships. Thus, given that team members in our experimental setup were strangers, our third hypothesis posited that higher levels of *attentional reciprocity* would positively affect their self-reported *team cohesion*.

**H3.** *Higher levels of attentional reciprocity within teams positively affect team cohesion.*

Detailed information on the statistical methods used to test our hypotheses is provided in the Supporting Information (see Supporting Information: S2 File and S3 Table).

## Results and discussion

For an overview of the key variables’ summary statistics and pair-wise correlations pooled across both treatments, see Supporting Information: Supporting Information S5 Table.

### Attentional reciprocity

**Fig 4:**
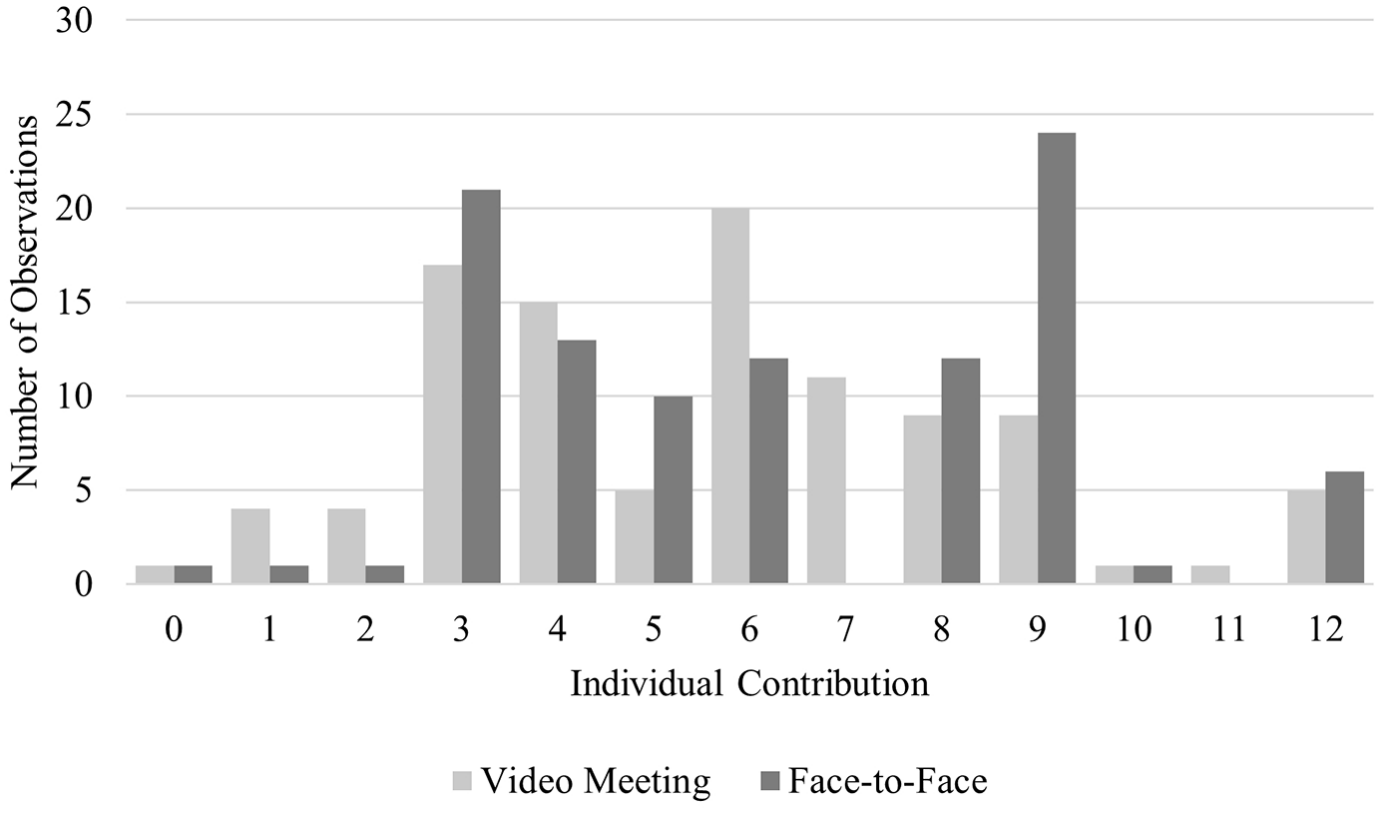
Distribution of Individual Contributions. Histogram showing the number of observations at each *individual contribution* level, displayed separately by treatments.

In both treatments, individual participants (*n* = 204) predominantly gazed at the task (*M* = 74.69, *SD* = 16.14) during the pre-play communication stage, followed by the AOIs of their team members (*M* = 19.22, *SD* = 13.11), and elsewhere (*M* = 6.09, *SD* = 6.62). Furthermore, participants allocated significantly more gaze to their team members in the *FT* (*M* = 21.62, *SD* = 14.30) compared to *VT* (*M* = 16.82, *SD* = 11.36), *t*(202) = 2.65, *p* = .009, *d* = 0.37, and significantly less gaze to areas outside the defined AOIs in *FT* (*M* = 5.06, *SD* = 5.15) than *VT* (*M* = 7.13, *SD* = 7.70), *t*(202) = 2.25, *p* = .026, *d* = 0.32 (see Table 1). Thus, the option to perceive the focus of gaze in *FT* helped team members to coordinate their gaze and to attend to each other more frequently. Overall, *joint attention* (i.e., individuals are gazing at the same object or aspect within the visual scene) was the most prevalent among social gaze dynamics within teams (*n* = 68, *M* = 58.09, *SD* = 20.03), succeeded by *gaze aversion* (*M* = 26.77, *SD* = 12.67) and *mutual gaze* (*M* = 8.03, *SD* = 7.98; see Fig 4).

**Table 1:** Proportional distribution of gaze between areas of interests.

	Video Meeting (*n* = 102)	Face-to-Face (*n* = 102)			
**Variable**	***M* (*SD*)**	***M* (*SD*)**	***t*(202)**	***p***	**Cohen’s *d***
Members	16.82 (11.36)	21.62 (14.30)	2.65	<.009***	0.37
Task	76.06 (15.28)	73.32 (16.91)	1.21	.227	0.17
Else	7.13 (7.70)	5.06 (5.15)	2.25	.026**	0.32

* p < 0.10, ** *p* < 0.05, *** *p* < 0.01.

Whereas *mutual gaze* was significantly higher in *FT* than *VT* (*p* < .001), we observed no significant differences in *gaze aversion* (*p* = .197) and *joint attention* (*p* = .797) between

the treatments (see Table 2). Overall, our results suggest that the informational value of gaze in *FT* was essential for maintaining comparatively higher levels of *mutual gaze*. Moreover, these findings indicate that the level of *gaze aversion* and *joint attention* did not depend on the option to perceive the focus of gaze during communication. Given that *FT* had a positive effect on *mutual gaze*, while *gaze aversion* remained constant across treatments, we also found significantly higher levels of *attentional reciprocity* (i.e., $\frac{Mutual\;Gaze}{Mutual\;Gaze\;+\;Gaze\;Aversion}$) in *FT* than *VT* (*p* < .001; see Table 2).

**Table 2:** Treatment differences in social gaze dynamics based on teams.

	Video Meeting (*n* = 34)	*F*ace-to-*F*ace (*n* = 34)			
**Variable**	***M* (*SD*)**	***M* (*SD*)**	***t*(66)**	***p***	**Cohen’s *d***
Mutual Gaze	4.78 (4.52)	11.29 (9.34)	3.66	<.001***	0.89
Gaze Aversion	28.77 (13.86)	24.78 (11.21)	1.30	.197	0.32
Joint Attention	57.46 (21.15)	58.72 (19.13)	0.26	.797	0.06
Attentional Reciprocity	11.51 (8.02)	26.37 (14.57)	5.21	<.001***	1.26
Team Output	17.03 (6.83)	18.65 (6.98)	0.97	.338	0.23
Team Cohesion	16.62 (2.05)	16.19 (2.38)	0.78	.437	0.19

* *p* < 0.10, ** *p* < 0.05, *** *p* < 0.01.

Testing our first hypothesis (H1), the first stage of two-stage least squares (2SLS) regressions provided strong evidence for the relevance of our experimentally randomized treatment variable *face-to-face*, *F*(2,65) = 43.66, *p* < .001 (see Table 3). Note that we decided to include *joint attention* in the second model due to several reasons. First, our initial results did not indicate any treatment effects on the level of *joint attention* (*p* = .797). Hence, adding *joint attention* as a control variable in the first stage did not add endogeneity bias to the estimated effect of *attentional reciprocity* in the second model. Second, the level of *joint attention* was inherently correlated with *attentional reciprocity*, as they are mutually exclusive dynamics (see Supporting Information: S5 Table). Accordingly, to avoid omitted variable bias stemming from this shared variance, we added *joint attention* to the model. Moreover, a large body of literature demonstrates a close link between *joint attention* and collective performance, making it a highly relevant variable to control for in team settings (for a review, see [64]).

**Table 3:** 2SLS Regression Analyses.

Variable	Coef.	*SE*	*p*
	**1**^**st**^ **Stage predicting Attentional Reciprocity:** R^2^ = 0.528; *F*(2,65) = 43.66, *p* < .001		
Face-to-Face	15.292	2.334	<.001
Joint Attention	-0.337	15.106	<.001
Constant	30.867	4.025	<.001
	**2**^**nd**^ **Stage predicting Team Output:** R^2^ = 0.152; χ^2^(2) = 8.88, *p* = .012		
Attentional Reciprocity	0.098	0.101	.332
Joint Attention	0.132	0.046	.004
Constant	8.314	4.132	.044
	**2**^**nd**^ **Stage predicting Team Cohesion:** R^2^ = 0.000; χ^2^(2) = 0.61, *p* = .738		
Attentional Reciprocity	-0.028	0.036	.442
Joint Attention	-0.008	0.018	.669
Constant	17.384	1.622	<.001

*n* = 68; reported standard errors are robust.

Our findings show that the coordination of attentional processes between team members was effectively impaired by our treatment manipulation. As expected, the absence of the ambiguity of gaze in *VT* has led to a substantial decrease in *attentional reciprocity*. Furthermore, the results revealed that our experimentally randomized treatment variable *face-to-face* satisfied the criteria for a valid instrument in 2SLS estimation. First, it met the relevance criterion, evidenced by the significant first-stage *F*-statistic, *F*(2,65) = 43.66, *p* < .001, which indicates a strong predictive value for *attentional reciprocity*. Second, in line with the exclusion restriction, we designed *face-to-face* to influence *team output* and *team cohesion* only through its impact on *attentional reciprocity*. However, one might argue that the key difference between face-to-face and video meetings lies not only in the perception of social gaze but more fundamentally in the physical distance between team members. This spatial separation could influence perceived social presence, thereby introducing an alternative pathway through which the treatment might affect outcomes, potentially violating the exclusion restriction. To address this concern, we conducted two-sided t-tests across six distinct dimensions of social presence [52] and found no evidence of a treatment effect on any of these dimensions (see Supporting Information: S6 Table). Finally, given that we randomly assigned teams to either *FT* or *VT*, the random assignment criterion was naturally supported. Therefore, we continued to calculate the second-stage regressions with *team output* and *team cohesion* regressed on the instrumented level of *attentional reciprocity* (see Table 3; for a more detailed overview, see Supporting Information: S7 Table).

#### Cooperation.

Overall, we observed a medium level of cooperation as *team output* averaged 17.84 pieces (*n* = 68, *SD* = 6.90, Min = 3, Max = 36), with only nine teams successfully maximizing team surplus. The most frequent decision made by individual participants (*n* = 204, *M* = 5.95, *SD* = 2.80, Min = 0, Max = 12) was an *individual contribution* of three pieces (*n* = 38; see Fig 4). This aligned with the Nash equilibrium ($x_{i}^{*}$ = 3), which risk-neutral, money-maximizing participants were predicted to choose. Notably, a large share of participants also selected to contribute nine pieces (*n* = 33), which would have maximized team surplus if chosen by all members ($x_{i}^{TS}$ = 9). Whereas *individual contributions* consistent with the Nash equilibrium were approximately equally frequent between *FT* (*n* = 21) and *VT* (*n* = 17), 73% of participants who opted for the team surplus maximizing decision belonged to *FT*. Thus, if a participant decided to deviate from purely selfish money-maximizing behavior, the extent of that deviation was higher after pre-play communication in *FT* than in *VT*. As an exploratory (non-preregistered) analysis, we also compared overall *team output* between treatments. *Team output* was higher in *FT* (*M* = 18.65, *SD* = 6.83) than in *VT* (*M* = 17.03, *SD* = 6.98). This difference, however, was not statistically significant, *t*(66) = 0.97, *p* = .338 (see Table 2).

To test the second hypothesis (H2), we proceeded with our 2SLS estimation strategy. Accordingly, we computed the second stages of the 2SLS models including *joint attention* as a control variable, then tested for endogeneity, and finally compared our results to those obtained using ordinary least squares (OLS) as an efficient estimator. Contrary to H2, second-stage 2SLS regressions revealed no significant effects of the instrumented level of *attentional reciprocity* (*p* = .332; see Table 3).

We took advantage of 2SLS estimation to address endogeneity concerns regarding the level of *attentional reciprocity* within teams and, thus, to examine its causal relationship with *team output*. However, post-hoc testing for endogeneity using Wooldridge’s robust scores revealed strong evidence that we did not face a problem with endogeneity (χ² = 0.524, *p* = .469). Following established guidelines for 2SLS estimation [65,66], we therefore re-estimated the equations using OLS.

In support of H2, OLS regressions indicated a significant positive relationship between *attentional reciprocity* and *team output* in the second model (*p* = .031; see Table 4). Notably, however, this effect was only significant when *joint attention* was included as a control variable. As previously discussed, this could be attributed to the fact that *joint attention* and *attentional reciprocity* are mutually exclusive dynamics and, thus, were inherently correlated. Therefore, estimating the effects of *attentional reciprocity* without controlling for *joint attention* would have introduced omitted variable bias. Additionally, *joint attention* contributed valuable information to the model, as highlighted by the increase in explained variance from approximately 1% in the first model, *F*(1,66) = 0.63, *p* = .431, to about 16% in the second model, *F*(2,65) = 7.16, *p* = .002. Yet, when controlling for *face-to-face* and its interaction with *attentional reciprocity* in the third model, the positive relationship between *attentional reciprocity* and *team output* was rendered insignificant (*p* = .684; see Table 4). This shows that in *VT*, where gaze information was ambiguous, *attentional reciprocity* did not positively impact *team output*. Notably, however, two-sided *F*-tests of the linear hypothesis *attentional reciprocity* +face-to-face*attentional reciprocity revealed that in *FT*, where gaze was informative, *attentional reciprocity* showed a significant positive relationship with *team output*, *F*(1,63) = 4.59, *p* = .036. These findings highlight that simply attending to each other in *VT* was not sufficient to establish successful cooperation. For *attentional reciprocity* to enhance cooperation within teams, the focus of gaze must be recognizable. Consistent with this, previous studies on social gaze showed that speakers tend to divide their gaze evenly among listeners, likely to assess whether they are paying attention during communication. Given that *attentional reciprocity* only showed an effect on *team output* in the *FT*, we conducted additional OLS regressions separated by treatments and included further controls such as *female* (0 = male, 1 = female) and *team cohesion* as further robustness checks. However, the results of these treatment-specific regressions did not reveal a consistent positive effect of *attentional reciprocity* in either setting (see Supporting Information: S8 Table).

**Table 4:** OLS Regressions with Team Output.

Variable	(1) Team Output	(2) Team Output	(3) Team Output
Attentional Reciprocity	0.055 (0.069)	0.157** (0.071)	0.075 (0.184)
Joint Attention		0.151*** (0.041)	0.157*** (0.049)
Face-to-Face			-3.566 (3.097)
Face-to-Face* Attentional Reciprocity			0.146 (0.190)
Constant	16.805*** (1.377)	6.067* (3.146)	7.129* (4.068)
R^2^	0.012	0.163	0.181
*F*-statistic	0.63	7.16	3.52
*p*-value	.431	.002	.012
Observations	68	68	68

Robust standard errors in parentheses * *p* < 0.10, ** *p* < 0.05, *** *p* < 0.01.

In summary, our 2SLS regressions did not reveal a causal relationship between *attentional reciprocity* and *team output*. Consequently, we rejected our second hypothesis (H2), positing that *attentional reciprocity* would universally promote team cooperation across different meeting modes. However, the results of our OLS regressions provided initial evidence that *attentional reciprocity* might serve as a key signal for type detection in face-to-face communication. Aligning with [15], this type detection mechanism appeared vital for successful team cooperation. Thus, future research should further investigate the contextual factors and underlying processes that enable *attentional reciprocity* to enhance cooperative behavior, particularly in computer-mediated communication, where the informative value of gaze information is missing.

### Team cohesion

In general, we observed high levels of *team cohesion* (*n* = 68, *M* = 16.41, *SD* = 2.21, Min = 8.94, Max = 19). However, in contrast to the cohesion-performance literature [2,34], we found no significant relationship between *team cohesion* and *team output* (*p* = .309; see Supporting Information: S4). Moreover, an exploratory (non-preregistered) comparison revealed that self-reported levels of *team cohesion* were similar between *FT* (*M* = 16.19, *SD* = 2.38) and *VT* (*M* = 16.62, *SD* = 2.05), with no statistically significant difference, *t*(66) = 0.78, *p* = .437 (see Table 2).

Contrary to our third hypothesis (H3), second-stage 2SLS regressions revealed no significant effect of *attentional reciprocity* on *team cohesion* (*p* = .442; Table 3 and Supporting Information: Table S3). When testing for endogeneity in a further step, Wooldridge’s robust scores indicated a high likelihood of endogeneity (χ² = 5.008, *p* = .025), suggesting that *attentional reciprocity* was indeed correlated with the error term when predicting *team cohesion*. Given that OLS does not account for the correlation between endogenous regressors and the error term, we did not compare our 2SLS estimation results with those obtained using OLS. Consequently, we rejected H3, proposing that higher levels of *attentional reciprocity* would positively impact *team cohesion*.

## Conclusions

Our findings provide valuable insights into the role of nonverbal signals in team communication, the relationship between social gaze dynamics and (non-)cooperative behavior, and the potential negative impact of ambiguous gaze information in computer-mediated communication. Unlike previous studies, our multiparty eye-tracking setup allowed us to capture participants’ eye movements during communication in face-to-face and video meeting settings with three participants. This enabled us to detect differences in *attentional reciprocity* between these two settings and examine their effects on *team output* and *team cohesion*.

Supporting our first hypothesis, H1, we found that *attentional reciprocity* within teams differed significantly between face-to-face and video meeting settings. Whereas direct gaze was reciprocated about 11% of the time in the video meeting setting, this likelihood more than doubled to around 26% when team members communicated face-to-face. This result indicates that the ambiguity of gaze in video settings impairs the ability of team members to coordinate their visual attention toward each other. Importantly, our findings suggest that the ability to observe what others are gazing at plays a key role in maintaining mutual gaze, rather than in coordinating attention to the task itself. While joint attention to task-related elements remained stable across both settings, mutual gaze was significantly reduced in video meetings. This implies that gaze serves not only a functional role in guiding task coordination but also a social role in signaling mutual attention and engagement among team members in face-to-face settings. This insight challenges a prevailing focus in design science research. To date, most technological interventions addressing gaze ambiguity in video meetings have focused on shared gaze visualizations to support joint attention during collaboration (for a review, see [67]). However, our findings suggest that the level of joint attention does not differ between face-to-face and video meeting settings. Rather, it is mutual gaze that is compromised. Despite this, very few systems have been developed to support mutual gaze in video meetings [68,69], highlighting a critical gap in their design. To better support the coordination of attentional processes within teams, future systems should incorporate ways of visualizing gaze information that helps team members remain visually connected not just to the task, but to each other.

Regarding our second hypothesis, H2, 2SLS estimation did not reveal that higher levels of *attentional reciprocity* universally promoted cooperation across both face-to-face and video meeting settings. However, OLS regressions provided a more detailed perspective. Consistent with the 2SLS results, we observed no significant relationship between *attentional reciprocity* and *team output* when communication took place via video meetings. Conversely, when individuals had the option observe each other’s focus of gaze during face-to-face communication, higher levels of *attentional reciprocity* were significantly associated with greater *team output*. This context-dependent pattern suggests that frequently attending to one another is not sufficient for successful cooperation, but whether this mutual attention to each other is recognizable during communication. In face-to-face settings, individuals can directly perceive when others attend to them, allowing the level of *attentional reciprocity* to function as a social signal. Given that visual attention is a limited and inherently costly resource (for a review, see [70]), noticing that it is reciprocated within the team may be interpreted as deliberate and prosocial, thereby fostering subsequent cooperative behavior. In video meetings, however, the ambiguity of gaze prevents team members from perceiving the level of *attentional reciprocity*. As a result, it cannot serve as a social signal, which may explain why we observed no significant relationship between *attentional reciprocity* and *team output* in the video meeting setting. More broadly, this finding aligns with prior research showing that individuals use gaze information not only to guide their own actions but also to evaluate the intentions and prosocial motives of others [10,11]. Similar findings in psychology show that the perception of eye contact can foster interpersonal trust, reinforcing the idea that gaze carries important social meaning [63]. Furthermore, this interpretation aligns well with the findings of [15], who demonstrate that conditional cooperators rely on nonverbal signals, in addition to verbal ones, to assess whether others are likely to act cooperatively. In this sense, *attentional reciprocity* in face-to-face settings may serve as a type detection mechanism, helping individuals identify cooperative partners and adjust their behavior accordingly.

Finally, we observed no effect of *attentional reciprocity* on *team cohesion*, which contradicted our third hypothesis, H3. Consequently, while cooperative behavior may indeed be influenced by the extent to which direct gaze is reciprocated within teams, the formation of *team cohesion* is not affected.

### Limitations & future research

The implementation of our novel multiparty eye-tracking setup yielded valuable insights into the role of social gaze dynamics in teams, but also introduced certain methodological limitations. The controlled meeting setups, while necessary to ensure precise and comparable measurements, diverged from naturalistic team settings. For example, the face-to-face setting included physical barriers with cutouts that allowed participants to only see each other’s upper half, creating an artificial environment compared to typical on-site meetings, where individuals share an open space. Similarly, the video meeting setup displayed each team member on separate monitors to ensure consistent and equal visibility among participants. While this configuration allowed for controlled comparisons between face-to-face and video meeting settings, it did not fully replicate the layout of conventional video meeting systems, where participants are typically displayed together on a single screen.

Another limitation concerns the potential influence of the eye-tracking equipment itself. Although the Pupil Invisible glasses are specifically designed to be lightweight and unobtrusive—closely resembling conventional eyewear—subtle effects of being observed or of observing others wearing eye-tracking devices, cannot be ruled out. Similarly, the reliance on QR codes to track the defined AOIs added another layer of artificiality to the setup. Although this method was a practical and necessary solution for accurately quantifying social gaze dynamics, it may have limited the generalizability of the findings. Recently, advancements in AI-driven eye-tracking technologies enabled the identification of AOIs without relying on QR-based image recognition, paving the way for more naturalistic experimental designs [53]. Thus, future studies employing such methods could preserve methodological rigor while enhancing the ecological validity of findings, offering deeper insights into the role of social gaze dynamics in real-world team setting.

Moreover, our design choice to facilitate open communication, recognizing gaze as a facet of multimodal interaction [28], may have inadvertently introduced systematic differences in communication content between our treatments. These differences could have influenced participants’ visual attention—specifically, whether they focused on the task or on each other—thereby shaping patterns of *attentional reciprocity*. Thus, future research should explore the interplay between communication content and gaze behavior.

Beyond these design limitations, we could not establish causal conclusions about the relationship between *attentional reciprocity* and team cooperation, as 2SLS estimation did not reveal significant effects. Although this approach is generally effective in testing causality, it did not allow for differentiating effects between our treatments. In contrast, OLS regressions provided this distinction, indicating that social gaze dynamics during face-to-face communication were associated with subsequent cooperative behavior. Based on these findings, future research could prioritize measuring social gaze dynamics during face-to-face communication, where gaze information is naturally available, to evaluate whether higher levels of *attentional reciprocity* act as a signal for prosocial motives and, in turn, promote cooperation. To achieve this, however, researchers need to develop an instrumental variable for 2SLS estimation that directly influences the level of *attentional reciprocity* without restricting access to gaze information. This approach would allow for a more precise evaluation of its role in fostering cooperative behavior while eliminating confounding factors arising from differences between meeting settings.

An alternative direction for future research could involve exploring the causal effects of social gaze dynamics in remote settings, where gaze information is naturally limited. Video meeting systems offer a unique opportunity to test whether introducing or enhancing the visibility of social gaze dynamics influences cooperative behavior within a controlled environment, rather than comparing face-to-face and computer-mediated communication. For example, a video meeting system could be designed to visualize episodes of bidirectional direct gaze between team members, allowing participants to observe and adjust their behavior according to the level of *attentional reciprocity*. Comparing the effects of such a system with a baseline—representing a conventional video meeting system—could offer valuable insights into the role of social gaze dynamics in enhancing team cooperation. In particular, it would deepen our understanding of whether and how the option to observe the level of *attentional reciprocity* functions as a signal of prosocial motives in team setting.

## Supporting information

S1 File Instructions. Instructions of the study.
(DOCX)

S2 File Estimation Strategy. (DOCX)

S3 Table Two-Stage Least Squares (2SLS). (DOCX)

S4 Fig Areas of Interest. (DOCX)

S5 Table Summary Statistics. (DOCX)

S6 Table Treatment Differences in Social Presence. (DOCX)

S7 Table Detailed Overview of 2SLS Regressions. (DOCX)

S8 Table OLS Regressions with Team Output separated by Treatments. (DOCX)
