# Supplementary material for: Social gaze dynamics in teams: Comparing face-to-face and video meeting settings
Source: PLoS One. 2026 Mar 2;21(3):e0329060. doi: 10.1371/journal.pone.0329060 (PMC12952598; doi:10.1371/journal.pone.0329060)
Supplement: S1 File — Instructions of the study. (DOCX) [file pone.0329060.s001.docx]

S1: Instructions

**Welcome,**

before we start, we would like to give you some essential information about our study.

In the context of the study, we use the currency “ECU”. This will be converted into Euros for your compensation at the end of the study. Thereby, 1.00 € equals 10 ECU.

At the beginning of the study, you will receive an endowment of 72 ECU. The amount of your payoff depends on your decision as well as the decisions of your team members in the experimental team task. You will be informed of the exact amount of your payoff in two weeks.

Participation in the study takes about 45 minutes. Please take your time when answering the questions.

Please click on the continue button to start. There is no possibility to return to a previous page. Therefore, please answer all questions in the given order.

**__________________________________________________**

**Thank you for completing the pre-survey**,

before we continue, we would like to describe the procedure of the main part of this study.

In the main part, you will work with two other participants to solve a team task. The goal of the team task is to create a unique shape based on geometric pieces. To do this, you will be given access to an individual computer where you can work together with your team members in a virtual workspace.


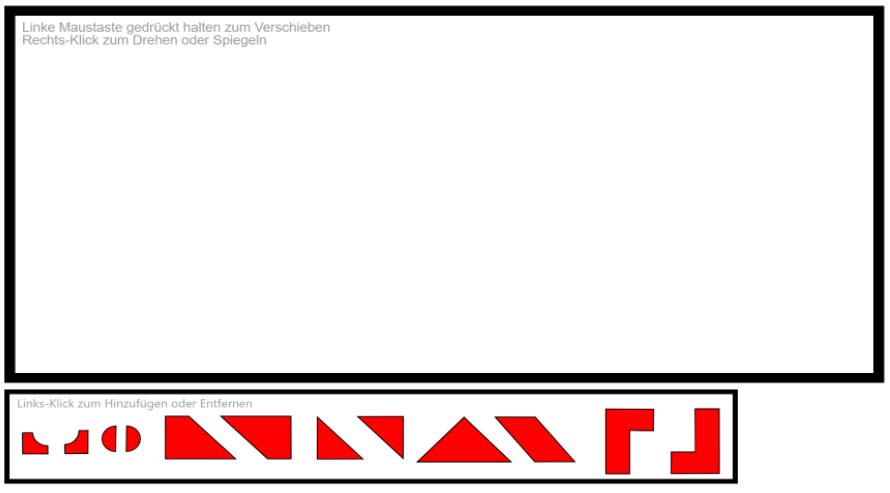


The main part is divided into two phases.

1) Practice phase: First, we want to make sure that you gain a basic understanding of how to work on the team task. For this purpose, you will receive a detailed explanation about the objective and the rules of the team task. We will also describe in detail what will affect the amount of your payoff.

2) Team task: After the practice phase, you will be equipped with eye-tracking glasses and guided to the room where the team task will be performed. After completing the team task with your team members, you will be guided back to the individual cubicle you are currently in.

**__________________________________________________**

**Tangram puzzle**

The team task is based on a tangram puzzle. A tangram is a set of building blocks in geometric shapes. These building blocks can be used to create various shapes reminiscent of animals or objects.

The tangram puzzle has no particular rules. For example, the building blocks do not necessarily have to touch each other. Thus, figures can also include distinct components.

**__________________________________________________**

**Instructions**

Below you will receive detailed information about the team task as well as the payoff principle. Please take your time to understand the objective and the rules of the game.

**Team task**

Once the team task begins, you will be equipped with eye-tracking glasses and led to another room.

*[If treatment = face-to-face]* In this room, you will be asked to take a seat at a table with a computer. The table is separated from those of your team members by partitions. However, the partitions contain rectangular cut outs so that you can see and communicate with your team members despite being spatially separated.

*[If treatment = video meeting]* In this room, you will be asked to take a seat at a table with a computer and three monitors. While performing the team task on the central monitor, your team members will be displayed on the left and right monitor via video meetings so that you can see and communicate with your team members despite being spatially separated.

You will have 5 minutes to complete the team task. At the end of the 5 minutes, you will be guided back to your individual cubicle.

The team task is based on the tangram puzzle, which was introduced on the previous page. In the team task, you will be given access to 12 tangram building blocks within a virtual workspace. Together with your team members, you will have a total of 36 building blocks to complete the task.

The objective of the team task is to work together with your team members to create a shape from the available building blocks. You can talk with your team members during the 5 minutes and add as many blocks as you like into the shared area for creating the tangram. You can also remove your own blocks from the interactive workspace at any time. The blocks have different colors to make it easier to distinguish between team members.

Thus, the team task is divided into two phases:

1) Team phase: Within the 5 minutes, you and your team members need to agree on a tangram shape as well as the building blocks required for it.

2) Decision phase: After completing the team phase, you will be escorted back to your individual cubicle to make a final decision about how many of your 12 building blocks you would like to contribute to the team task.

Your individual decision will not be disclosed to the other participants and will therefore be anonymous. Likewise, you will not be informed of the individual decisions of your team members.

Your individual decision and the decisions of your two team members will determine the amount of your payoff.

**Payoff**

To complete the team task, each team member receives a set of 12 tangram blocks. Thus, a total of 36 blocks will be available to create a shape. Each team member decides individually on the number of pieces they want to contribute to the team task.

The amount of your payoff depends on two factors.

**1) Individual revenue:** First, the sum of the building blocks contributed by all three team members is calculated. This sum can be between 0 and 36, since all three participants can contribute between 0 and 12 building blocks. The sum of the building blocks is then multiplied by 18 to determine the team’s overall revenue. In the second step, the team revenue is divided equally between the team members, so that each participant receives one third as individual revenue.


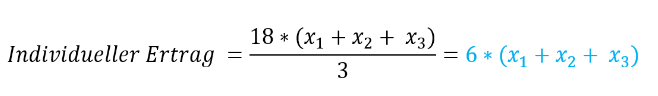


**2) Individual costs:** Each building block you contribute to the team task incurs a cost to yourself. These individual costs increase with each block you have already contributed and are deducted from your individual revenue.


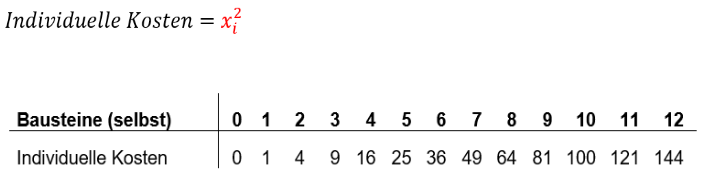


In combination with your endowment of 72 ECU, your individual payoff is calculated as follows:


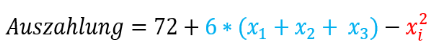


**__________________________________________________**

**Comprehension tasks**

Before we continue with the practice phase, we would like to ask you to complete the following comprehension tasks. For this purpose, please use the auxiliary materials provided at your workplace.

**1)** Please indicate the maximum number of building blocks you can personally contribute to the team task.

[Select option between 0 and 12] building blocks

*[If wrong]* Unfortunately, your answer to the question was not correct.

To complete the team task, each participant receives a set of 12 tangram building blocks.

**2)** Please indicate the maximum number of building blocks that your entire team can contribute to the team task.

[Select option between 0 and 12] building blocks

*[If wrong]* Unfortunately, your answer to the question was not correct.

To complete the team task, each participant receives a set of 12 tangram building blocks. Thus, each team receives a total of 36 building blocks to complete the task.

**3)** Please select all decisions that influence your individual revenue.

[] Your individual decision

[] Decisions of the other two participants

*[If wrong]* Unfortunately, your answer to the question was not correct.

Both your individual decision and the decisions of the other two participants influence your individual revenue, since it is based on the total number of building blocks contributed (0 to 36).

**4)** Please select all decisions that influence your individual costs.

[] Your individual decision

[] Decisions of the other two participants

*[If wrong]* Unfortunately, your answer to the question was not correct.

Only your individual decision influences your individual costs, as it is based on your individual contribution (0 to 12).

**5)** Please select the option that correctly reflects the payoff principle of the team task.

[] Payoff = Individual Costs - Individual Revenue + 72

[] Payoff = (Individual Costs - 72) + Individual Costs

[] Payoff = (Individual Revenue + 72) + Individual Costs

[] Payoff = 72 + (Individual Revenue - Individual Costs)

*[If wrong]* Unfortunately, your answer to the question was not correct.

The payoff principle is: Payoff = 72 + (Individual Revenue - Individual Costs)

**__________________________________________________**

**Practice phase**

On the following page, you will have the opportunity to get familiar with the operation of the virtual workspace for working together on the team task. In the virtual workspace, which is not shared with your team members during the practice phase, you have access to 12 Tangram blocks.


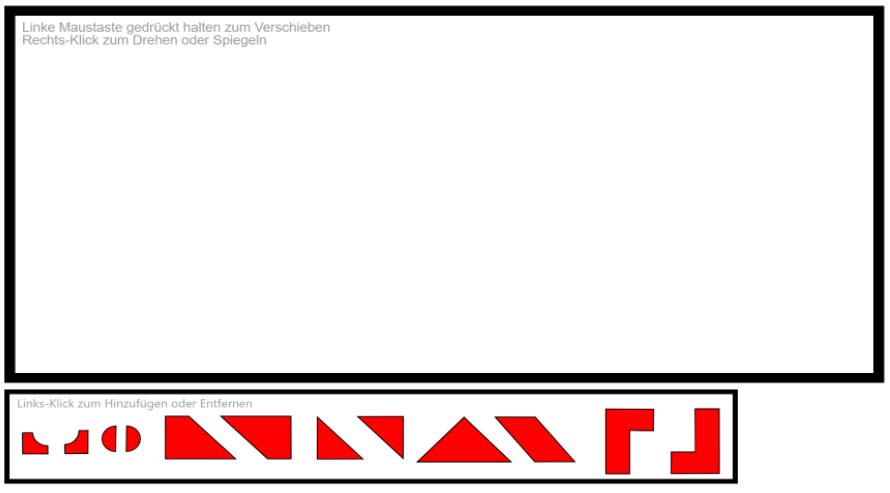


1) Left-click to add or remove: By clicking with the left mouse button (left-click), the blocks can be added to the larger area for creating and editing the tangram. You can also remove a block from the editing area by clicking on it again in the lower bar.

2) Hold down the left mouse button to move blocks: In the editing area, you can move the blocks by holding down the left mouse button (continued left-click).

3) Right-click to rotate or mirror: Furthermore, you can edit the alignment of the blocks in the editing area by clicking on them with the right mouse button (right-click). As soon as you have clicked on a block, you can rotate and mirror it around its own axis. Right-click on the block again to finish editing the alignment.

The number of building blocks used is neither observed nor recorded during the practice phase and thus has no influence on the payoff.

To continue, please click on the button at the bottom right corner of the screen as soon as the time on it has expired.

**__________________________________________________**

**Thank you for completing the practice phase,**

you have successfully completed the first part of the study. Next, the team task begins.

**Reminder**

The team task is divided into two phases.

1) During the 5 minutes, you and your team members need to agree on a shape to build as well as the building blocks required to achieve it. You can talk with your team members during the 5 minutes and add as many blocks as you like into the shared area for creating the tangram. You can also remove your own blocks from the interactive workspace at any time.

2) After the 5 minutes, you will be guided back to your individual cubicle. Next, you need to indicate how many of your 12 building blocks you would like to contribute to the team task.

Your individual decision and the decisions of your two team members in the second stage determine your payoff.

Please remain seated until you will be guided to the group room to complete the team task.

**__________________________________________________**

**Decision stage**

Please make a final decision on the number of building blocks you would like to contribute to the team task.

[Select option between 0 and 12] building blocks

**Reminder**

The amount of your payoff depends on two factors.

**1) Individual revenue:** First, the sum of the building blocks contributed by all three team members is calculated. This sum can be between 0 and 36, since all three participants can contribute between 0 and 12 building blocks. The sum of the building blocks is then multiplied by 18 to determine the team’s overall revenue. In the second step, the team revenue is divided equally between the team members, so that each participant receives one third as individual revenue.


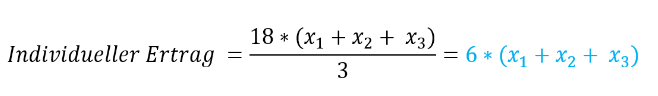


**2) Individual costs:** Each building block you contribute to the team task incurs a cost to yourself. These individual costs increase with each block you have already contributed and are deducted from your individual revenue.


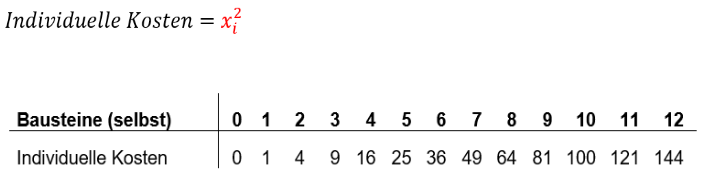


In combination with your endowment of 72 ECU, your individual payoff is calculated as follows:


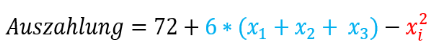


**__________________________________________________**

**Thank you for completing the team task,**

now please take your time to answer the questions included in the post-survey.

Please click on the continue button to start. There is no possibility to return to a previouspage. Therefore, please answer the questions in the given order.
