# Supplementary material for: Social gaze dynamics in teams: Comparing face-to-face and video meeting settings
Source: PLoS One. 2026 Mar 2;21(3):e0329060. doi: 10.1371/journal.pone.0329060 (PMC12952598; doi:10.1371/journal.pone.0329060)
Supplement: S2 File — (DOCX) [file pone.0329060.s002.docx]

S2. Estimation Strategy.

Main analyses were performed in Stata (v16.1; StataCorp., 2023). After pre-processing the data, we conducted an initial analysis to report the sample’s characteristics, pairwise correlations, and aggregated gaze patterns, including the distribution of gaze between AOIs (Members, Task, and Else; see Supporting Information: Fig S1) and the ratio of each respective social gaze dynamic.

We tested our hypotheses (**H1**, **H2**, and **H3**) using two-stage least squares regressions (for a comprehensive explanation of two-stage least squares estimation, see Supporting Information: Appendix S3) with the level of *attentional reciprocity* instrumented by our experimentally randomized treatment variable *face-to-face* (0 = *VT*, 1 = *FT*) and *team output* as well as *team cohesion* as dependent variables respectively. First, we examined if our experimentally randomized treatment variable *face-to-face* satisfied the (as if) randomness, exclusion, and relevance criterion to qualify as a valid instrument for the statistical testing of our hypotheses using 2SLS estimation (Bastardoz et al., 2023; Bound et al., 1995; Sajons, 2020; Wooldridge, 2010). Since we experimentally randomized teams to *FT* and *VT*, the (as if) randomness criterion was satisfied by design. Nevertheless, we performed a randomization check by calculating Pearson correlation coefficients between *face-to-face* and participants’ individual characteristics as part of the summary statistics.
