# Supplementary material for: Social gaze dynamics in teams: Comparing face-to-face and video meeting settings
Source: PLoS One. 2026 Mar 2;21(3):e0329060. doi: 10.1371/journal.pone.0329060 (PMC12952598; doi:10.1371/journal.pone.0329060)
Supplement: S3 Table — (DOCX) [file pone.0329060.s003.docx]

S3. Two-Stage Least Squares

**Definition.** Two-stage least squares (2SLS) estimation extends ordinary least squares (OLS) to address cases where the assumptions of OLS are violated. In particular, OLS assumes that all factors influencing $y$ are captured by the variation in $x$ and that $x$ is exogenous, meaning it is uncorrelated with the error term $\varepsilon$ (Wooldridge, 2010). When these assumptions hold, OLS provides unbiased and consistent estimates. However, when $x$ is endogenous, meaning it is correlated with the error term, OLS estimates become biased. The 2SLS method modifies the OLS approach by introducing instrumental variables to isolate the exogenous variation in $x$, thereby addressing endogeneity (Sajons, 2020).

The general form of the OLS regression is shown in Equation 1, where $y$ is the dependent variable, $x$ is the independent variable, $\beta_{0}$ is the intercept, $\beta_{1}$ ​is the coefficient of $x$, and $\varepsilon$ is the error term, representing all unobserved factors affecting $y$ (Bastardoz et al., 2023). For OLS to produce reliable estimates of $\beta_{1}$, $x$ must be exogenous. If $x$ is endogenous, the estimates will be biased and unsuitable for causal interpretation.

$y\text{=}\beta_{0}\text{+}\beta_{1}x\text{+}\varepsilon$ (Eq. 1)

Endogeneity, defined as the correlation between the explanatory variable and the error term, is a common issue in regression models (Bound et al., 1995; Wooldridge, 2010). It can arise due to omitted variables, sample selection bias, measurement error, or simultaneity/reverse causality.

**Procedure.** 2SLS estimation mitigates endogeneity through a two-stage process. In the first stage, the endogenous variable $x$ is regressed on an instrumental variable $z$ that is uncorrelated with the error term (Equation 2). This produces an estimated predictor $\hat{x}$, which captures only the exogenous variation in $x$.

$x\text{=}\delta_{0}\text{+}\delta_{1}z\text{+}u$ (Eq. 2)

In the second stage, $\hat{x}$ replaces $x$ in the original model, and $y$ is regressed on $\hat{x}$, as shown in Equation 3.

$y\text{=}\alpha_{0}\text{+}\alpha_{1}\hat{x}\text{ +}w$ (Eq. )
