# Supplementary material for: Social gaze dynamics in teams: Comparing face-to-face and video meeting settings
Source: PLoS One. 2026 Mar 2;21(3):e0329060. doi: 10.1371/journal.pone.0329060 (PMC12952598; doi:10.1371/journal.pone.0329060)

**Fig S4. Areas of Interest.** Areas of interest A, B, and Task, as seen from the visual perspective of team member C with heatmaps depicting the foci of visual attention. The upper panel shows the face-to-face and the lower panel the video set-up. The individuals in this figure have given written informed consent (as outlined in PLOS consent form) to publish their case details.


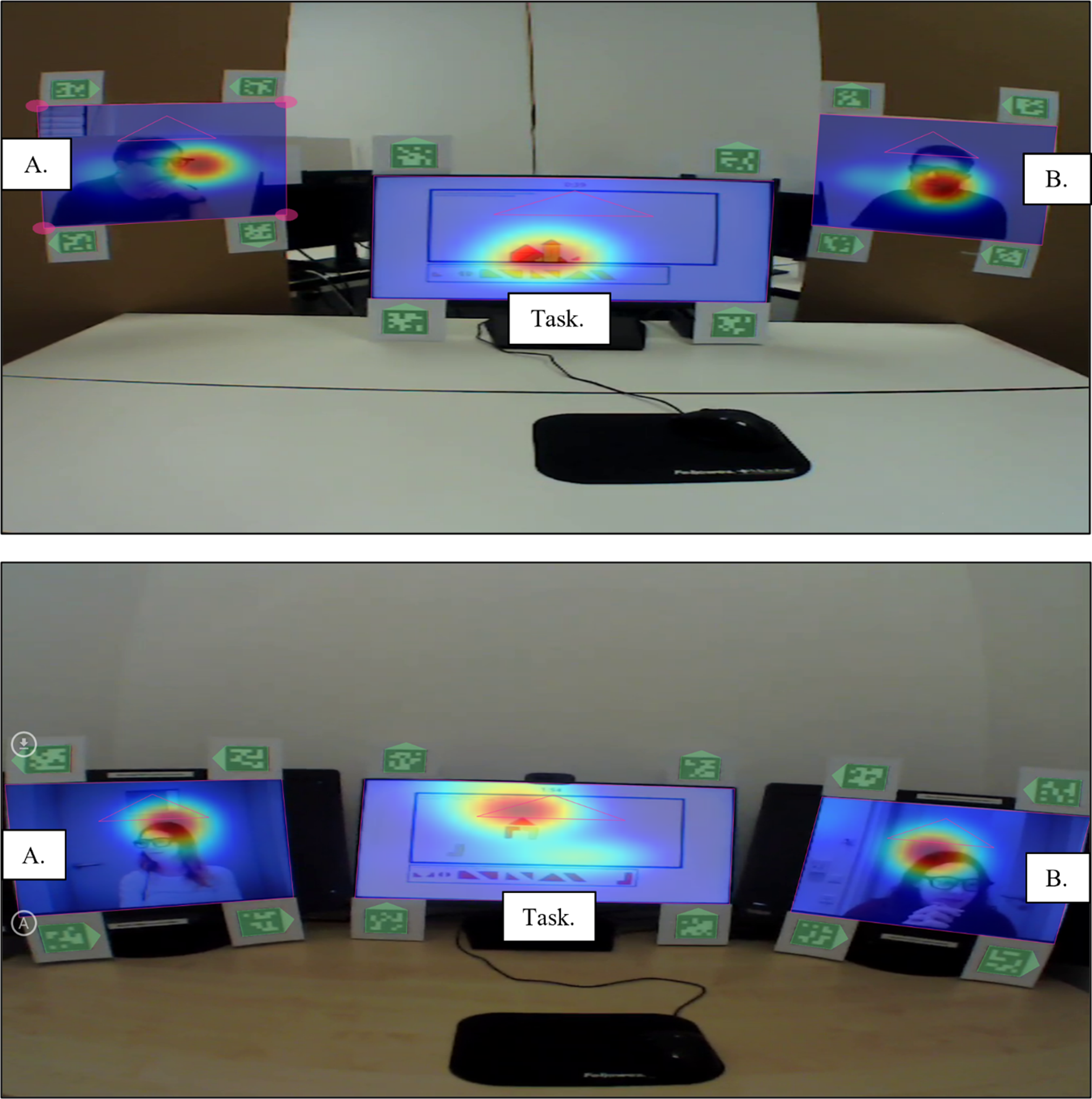

Supplement: S4 Fig — (DOCX) [file pone.0329060.s004.docx]
