# Supplementary material for: Social gaze dynamics in teams: Comparing face-to-face and video meeting settings
Source: PLoS One. 2026 Mar 2;21(3):e0329060. doi: 10.1371/journal.pone.0329060 (PMC12952598; doi:10.1371/journal.pone.0329060)
Supplement: S5 Table — (DOCX) [file pone.0329060.s005.docx]

**Table S5A. Summary Statistics at the Individual Level.**

| **Variable** | ***M*** | ***SD*** | **Min** | **Max** | **(1)** | **(2)** | **(3)** | **(4)** | **(5)** | **(6)** | **(7)** | **(8)** | **(9)** | **(10)** | **(11)** | **(12)** | **(13)** | **(14)** |
| --- | --- | --- | --- | --- | --- | --- | --- | --- | --- | --- | --- | --- | --- | --- | --- | --- | --- | --- |
| (1) Face-to-Face | 0.50 | 0.50 | 0.00 | 1.00 |  |  |  |  |  |  |  |  |  |  |  |  |  |  |
| (2) Female | 0.53 | 0.50 | 0.00 | 1.00 | -0.06 |  |  |  |  |  |  |  |  |  |  |  |  |  |
| (3) Age | 24.40 | 4.85 | 18.00 | 54.00 | 0.06 | -0.06 |  |  |  |  |  |  |  |  |  |  |  |  |
| (4) Individual Contribution | 5.95 | 2.80 | 0.00 | 12.00 | 0.10 | -0.09 | 0.10 |  |  |  |  |  |  |  |  |  |  |  |
| (5) Team Cohesion | 5.47 | 1.04 | 2.06 | 7.00 | -0.07 | 0.19*** | -0.03 | 0.21*** |  |  |  |  |  |  |  |  |  |  |
| (6) Belongingness | 4.74 | 1.60 | 1.00 | 7.00 | -0.05 | 0.10 | 0.02 | 0.25*** | 0.86*** |  |  |  |  |  |  |  |  |  |
| (7) Social Cohesion | 5.65 | 1.10 | 1.50 | 7.00 | -0.11 | 0.24*** | -0.07 | 0.10 | 0.86*** | 0.55*** |  |  |  |  |  |  |  |  |
| (8) Task Cohesion | 6.02 | 0.99 | 2.00 | 7.00 | -0.01 | 0.15** | -0.04 | 0.15** | 0.81*** | 0.48*** | 0.69*** |  |  |  |  |  |  |  |
| (9) Collective Orientation | 2.94 | 0.32 | 1.88 | 3.88 | 0.00 | 0.00 | 0.06 | 0.14** | 0.08 | 0.15** | 0.02 | 0.00 |  |  |  |  |  |  |
| (10) Interpersonal Trust | 3.12 | 0.45 | 1.67 | 4.33 | -0.01 | -0.01 | 0.00 | 0.03 | 0.15** | 0.08 | 0.17** | 0.18** | 0.03 |  |  |  |  |  |
| (11) Extraversion | 3.26 | 0.96 | 1.00 | 5.00 | -0.04 | 0.04 | 0.03 | -0.11 | 0.04 | 0.02 | 0.01 | 0.08 | -0.23*** | 0.03 |  |  |  |  |
| (12) Agreeableness | 3.33 | 0.83 | 1.00 | 5.00 | -0.04 | 0.18** | -0.05 | 0.14* | 0.17** | 0.10 | 0.21*** | 0.14* | 0.19*** | 0.27*** | 0.02 |  |  |  |
| (13) Conscientiousness | 3.58 | 0.83 | 1.50 | 5.00 | 0.02 | 0.04 | 0.03 | 0.02 | 0.13* | 0.11 | 0.14* | 0.09 | -0.05 | 0.02 | 0.10 | 0.00 |  |  |
| (14) Neuroticism | 3.00 | 1.01 | 1.00 | 5.00 | -0.04 | 0.38*** | -0.01 | 0.05 | 0.07 | 0.08 | 0.03 | 0.06 | 0.12* | 0.08 | -0.17** | 0.02 | 0.02 |  |
| (15) Openness | 3.51 | 1.08 | 1.00 | 5.00 | 0.05 | 0.23*** | 0.17** | -0.03 | 0.00 | -0.02 | 0.06 | -0.03 | -0.04 | -0.08 | 0.15** | -0.12* | 0.05 | 0.20*** |

^*^ *p* < 0.10, ^**^ *p* < 0.05, ^***^ *p* < 0.01, *n* = 204

**Table S5B. Summary Statistics at the Team Level.**

| **Variable** | ***M*** | ***SD*** | **Min** | **Max** | **(1)** | **(2)** | **(3)** | **(4)** | **(5)** | **(6)** | **(7)** | **(8)** |
| --- | --- | --- | --- | --- | --- | --- | --- | --- | --- | --- | --- | --- |
| (1) Face-to-Face | 0.50 | 0.50 | 0.00 | 1.00 |  |  |  |  |  |  |  |  |
| (2) Female | 0.53 | 0.50 | 0.00 | 1.00 | -0.06 |  |  |  |  |  |  |  |
| (3) Age | 24.40 | 2.76 | 20.67 | 34.33 | 0.10 | -0.10 |  |  |  |  |  |  |
| (4) Team Output | 17.84 | 6.90 | 3.00 | 36.00 | 0.12 | -0.11 | 0.14 |  |  |  |  |  |
| (5) Team Cohesion | 16.41 | 2.21 | 8.94 | 19.61 | -0.10 | 0.26** | -0.12 | 0.13 |  |  |  |  |
| (6) Attentional Reciprocity | 18.94 | 13.87 | 0.00 | 51.51 | 0.54*** | -0.05 | 0.00 | 0.11 | 0.14 |  |  |  |
| (7) Mutual Gaze | 8.03 | 7.98 | 0.00 | 33.49 | 0.41*** | -0.04 | -0.17 | 0.01 | 0.16 | 0.91*** |  |  |
| (8) Gaze Aversion | 26.77 | 12.67 | 2.76 | 56.03 | -0.16 | 0.04 | -0.30** | -0.36*** | 0.05 | 0.26** | 0.47*** |  |
| (9) Joint Attention | 58.10 | 20.03 | 14.66 | 96.10 | 0.03 | 0.01 | 0.28** | 0.29** | 0.01 | -0.47*** | -0.65*** | -0.91*** |

^*^ *p* < 0.10, ^**^ *p* < 0.05, ^***^ *p* < 0.01, *n* = 68
