# Supplementary material for: Social gaze dynamics in teams: Comparing face-to-face and video meeting settings
Source: PLoS One. 2026 Mar 2;21(3):e0329060. doi: 10.1371/journal.pone.0329060 (PMC12952598; doi:10.1371/journal.pone.0329060)
Supplement: S6 Table — (DOCX) [file pone.0329060.s006.docx]

**Table S6. Treatment Differences in Social Presence.**

|  | **Video Meeting** | **Face-to-Face** |  |  |  |
| --- | --- | --- | --- | --- | --- |
| **Variable** | ***M* (*SD*)** | ***M* (*SD*)** | ***t*(202)** | ***p*** | **Cohen’s *d*** |
| Co Presence | 6.00 (0.90) | 6.05 (0.77) | 0.39 | .697 | 0.05 |
| Attention Allocation | 5.17 (1.18) | 5.22 (1.07) | 0.27 | .787 | 0.04 |
| Message Understanding | 5.57 (1.10) | 5.69 (1.08) | 0.78 | .435 | 0.11 |
| Affective Understanding | 4.17 (1.30) | 4.35 (1.20) | 1.03 | .303 | 0.14 |
| Emotional Interdependence | 4.18 (1.22) | 4.34 (1.16) | 0.98 | .323 | 0.14 |
| Behavioral Interdependence | 5.04 (0.86) | 5.13 (0.91) | 0.73 | .468 | 0.10 |

*p* < 0.10, ^**^ *p* < 0.05, ^***^ *p* < 0.01; *n* = 204
