# Supplementary material for: Social gaze dynamics in teams: Comparing face-to-face and video meeting settings
Source: PLoS One. 2026 Mar 2;21(3):e0329060. doi: 10.1371/journal.pone.0329060 (PMC12952598; doi:10.1371/journal.pone.0329060)
Supplement: S7 Table — (DOCX) [file pone.0329060.s007.docx]

**Table S7A. First-Stage 2SLS Regressions.**

| **Variable** | **(1)**  **Attentional Reciprocity** | **(2)**  **Attentional Reciprocity** |
| --- | --- | --- |
| Face-to-Face | 14.886***  (2.853) | 15.292***  (2.334) |
| Joint Attention |  | -0.337***  (15.106) |
| Constant | 11.508***  (1.375) | 30.867***  (4.025) |
| R^2^ | 0.292 | 0.528 |
| *F*-statistic | 27.16 | 43.66 |
| *p*-value | <.001 | <.001 |
| Observations | 68 | 68 |

Robust standard errors in parentheses

^*^ *p* < 0.10, ^**^ *p* < 0.05, ^***^ *p* < 0.01

**Table S7B. Second-Stage 2SLS Regressions with Team Output.**

| **Variable** | **(1)**  **Team Output** | **(2)**  **Team Output** |
| --- | --- | --- |
| Attentional Reciprocity | 0.109  (0.112) | 0.098  (0.101) |
| Joint Attention |  | 0.132***  (0.046) |
| Constant | 15.777***  (2.294) | 8.314**  (4.132) |
| R^2^ | 0.000 | 0.152 |
| χ^2^-statistic | 0.95 | 8.88 |
| *p*-value | .330 | .012 |
| Observations | 68 | 68 |

Robust standard errors in parentheses

^*^ *p* < 0.10, ^**^ *p* < 0.05, ^***^ *p* < 0.01

**Table S7C. Second-Stage 2SLS Regressions with Team Cohesion.**

| **Variable** | **(1)**  **Team Cohesion** | **(2)**  **Team Cohesion** |
| --- | --- | --- |
| Attentional Reciprocity | -0.028  (0.037) | -0.028  (0.036) |
| Joint Attention |  | -0.008  (0.018) |
| Constant | 16.942***  (0.713) | 17.384***  (1.622) |
| R^2^ | 0.000 | 0.000 |
| χ^2^-statistic | 0.58 | 0.61 |
| *p*-value | .447 | .738 |
| Observations | 68 | 68 |

Robust standard errors in parentheses

^*^ *p* < 0.10, ^**^ *p* < 0.05, ^***^ *p* < 0.01
