# Supplementary material for: Social gaze dynamics in teams: Comparing face-to-face and video meeting settings
Source: PLoS One. 2026 Mar 2;21(3):e0329060. doi: 10.1371/journal.pone.0329060 (PMC12952598; doi:10.1371/journal.pone.0329060)
Supplement: S8 Table — (DOCX) [file pone.0329060.s008.docx]

**Table S8. OLS Regressions with Team Output separated by Treatments.**

|  | **Video Meeting** | | | | **Face-to-Face** | | | |
| --- | --- | --- | --- | --- | --- | --- | --- | --- |
| **Variable** | **(1)** | **(2)** | **(3)** | **(4)** | **(5)** | **(6)** | **(7)** | **(8)** |
| Attentional Reciprocity | -0.153  (0.184) | 0.128  (0.191) | 0.157  (0.212) | 0.183  (0.242) | 0.088  (0.089) | 0.177  (0.122) | 0.165  (0.126) | 0.129  (0.115) |
| Joint Attention |  | 0.194***  (0.057) | 0.197***  (0.060) | 0.207***  (0.070) |  | 0.104  (0.080) | 0.103  (0.081) | 0.102  (0.081) |
| Female |  |  | -0.928  (2.200) | -0.833  (2.198) |  |  | -1.283  (2.492) | -2.421  (2.654) |
| Team Cohesion |  |  |  | -0.243  (0.610) |  |  |  | 0.646  (0.468) |
| Constant | 18.789***  (2.155) | 4.435  (4.668) | 4.438  (4.796) | 7.546  (8.098) | 16.319***  (2.282) | 7.889  (7.354) | 8.875  (7.726) | 0.011  (11.044) |
| R^2^ | 0.032 | 0.282 | 0.286 | 0.290 | 0.034 | 0.081 | 0.089 | 0.129 |
| *F*-statistic | 0.69 | 6.50 | 4.16 | 3.20 | 0.99 | 1.13 | 0.82 | 0.79 |
| *p*-value | .412 | .004 | .014 | .027 | .327 | .337 | .492 | .543 |
| Observations | 34 | 34 | 34 | 34 | 34 | 34 | 34 | 34 |

Robust standard errors in parentheses

^*^ *p* < 0.10, ^**^ *p* < 0.05, ^***^ *p* < 0.01
